# Qualitative analysis of emotional distress in burns, plastic and reconstructive surgery patients from the perspectives of cognitive and metacognitive models

**DOI:** 10.3389/fpsyt.2024.1461387

**Published:** 2024-10-16

**Authors:** Joseph Taylor-Bennett, Lora Capobianco, Julie Wisely, Adrian Wells

**Affiliations:** ^1^ Manchester Specialist Psychotherapy Service, Greater Manchester Mental Health NHS Foundation Trust, Manchester, United Kingdom; ^2^ Division of Psychology and Mental Health, School of Health Sciences, University of Manchester, Manchester, United Kingdom; ^3^ Research and Innovation, Greater Manchester Mental Health NHS Foundation Trust, Manchester, United Kingdom; ^4^ Department of Clinical Psychology, Greater Manchester Mental Health NHS Foundation Trust, Manchester, United Kingdom

**Keywords:** cognitive-behaviour therapy, metacognitive therapy, burns, plastic and reconstructive surgery patients, emotional distress, qualitative research, cognitive attentional syndrome, cognitive distortions

## Abstract

**Introduction:**

Burns and other injuries requiring plastic and/or reconstructive surgery (BPRS) are lifechanging, often unexpected, and increase the risk of psychiatric morbidity. There are no published studies we are aware of that explores the applicability of psychological models to BPRS patients. Cognitive behavioural therapy (CBT) is the benchmark treatment in mental health but may be less effective in physical health settings. Metacognitive therapy (MCT)can be more effective than CBT in mental health settings and shows promise in reducing anxiety and depression symptoms in people with cancer and cardiac disease. The present study explored the psychological experiences (feelings, thoughts, and coping strategies) of BPRS patients, and whether the concepts underpinning cognitive and metacognitive models can be elicited from these accounts.

**Method:**

Semi structured interviews were conducted with 11 patients recruited from a BPRS psychology service. Data was analysed using Thematic Analysis. Patients described a range of emotions including low mood, anxiety, anger, guilt, loss, and negative thinking.

**Results:**

From the perspective of the cognitive model, there were examples of each of 10 pre-specified distorted thinking types (cognitive distortions), and patient talk seemed to fit problem-specific cognitive models. From the perspective of the metacognitive model, all patients described the “cognitive attentional syndrome,” i.e., how they engaged in repetitive negative thinking (worry, rumination) and thought-focused regulation strategies. Patient talk also demonstrated both positive and negative metacognitive beliefs.

**Conclusion:**

The implications of applying the findings from each model to clinical practice are discussed. The metacognitive model may offer benefits in clinical practice that should be investigated further.

## Introduction

Approximately 175,000 people attend Accident and Emergency departments and 16,000 people are admitted for specialist burn care each year in the UK (1). Additionally, 48,000 injuries are treated in major trauma centres (2), and over 5,000 women undergo breast reconstruction following mastectomy each year in England (3). Burns and other injuries that require plastic reconstructive surgery (BPRS) are associated with higher healthcare use, increased morbidity and mortality, and poorer quality of life (4, 5). Mental health conditions, such as depression, anxiety, and post-traumatic stress disorder (PTSD), are common after serious injuries and more prevalent in BPRS patients than in the general population (6, 7). Approximately 28% of burn injury patients received at least one psychiatric diagnosis post-injury (7), and prevalence of mental health disorders range from 30% to 70% after reconstructive surgery (8–11).

The National Burn Care Review Committee (1) highlights the need for routine screening and psychological support as part of aftercare, but there are currently no specific recommendations for treatment beyond the general problem-specific guidelines. Cognitive Behavioural Therapy (CBT) is an umbrella term for psychological therapies based on the idea that thoughts, feelings, and behaviours are interconnected, and that changing maladaptive thinking patterns can lead to changes in emotions and behaviours. They have their theoretical basis in cognitive theory (12–14). Applications of this theory and an integration with behavioural approaches have resulted in a multitude of problem-specific models, which have become the most widely recommended and effective treatments for mental health conditions, including depression, anxiety, and PTSD (15–17). The cognitive theory that underpins much of these models states that negative automatic thoughts (NATs) are triggered by situations and events and are anchored in beliefs that individuals hold about themselves, other people, and the world around them. These beliefs are influenced by past experiences and can either be intermediate rules and assumptions about how to live or entrenched core beliefs. Cognitive distortions are evident in NATs and core beliefs and represent biased patterns of thinking that maintain negative interpretations and beliefs about the self and world. A principal focus of cognitive therapy based on this model is to identify and modify the content of NATs through challenging their validity, modifying cognitive distortions, and reality-testing beliefs. The many and varied ways in which CBT has been adapted and applied to specific problems can make interpretations of research that claims it difficult to apply as they may have different models and differing emphases on cognitive and behavioural components. There are also a multitude of “third-wave” CBT interventions, such as Dialectical Behaviour Therapy (DBT; 18), Compassion Focussed Therapy [CFT; (19)], and Acceptance and Commitment Therapy [ACT; (20)] to name a few. These, however, are often more clearly distinguished within research as they deviate more substantially from Beck’s original cognitive model. Thus, while it is important to note that not all CBT uses the same protocol, much of the research that claims to use CBT more often than not holds the cognitive model at the core of its approach.

While CBT has not been trialled with BPRS patients specifically, the effect size in physical illnesses was shown to be small for both anxiety (*d* = 0.34) and depression (*d* = 0.35) in recent meta-analyses (21, 22). The mechanisms underlying lower efficacy in physical health compared to mental health is currently unknown. One possible explanation is that the cognitive model may not address all distressing NATs that are clinically relevant to this population; “classic CBT techniques, such as cognitive restructuring, are inadequate or even inappropriate for patients with realistic fears related to the cancer diagnosis and treatment” (23, p. 3). Similarly, McPhillips et al. (24) conducted a qualitative analysis of the emotional distress described by cardiac patients who screened positive for symptoms of depression or anxiety. They found that whilst, CBT offered a framework that could be utilised in formulating patient distress, there were challenges in categorising some distressing thoughts as realistic or not.

An alternative approach to treating psychopathology that does not rely at all on how realistic thoughts are, is Metacognitive Therapy [MCT; (25)]. MCT is based on the metacognitive model (25–27) where psychological disorder is caused by biases in mental regulation that appear as a “cognitive attentional syndrome” of dysregulated worry, rumination, and attention processes. Such a syndrome is linked to the activity of a metacognitive control system that is separate from cognition but guides the latter under the influence of metacognitive beliefs. In a meta-analysis of MCT trials within mental health, the results appear to favour MCT over CBT in reducing symptoms of anxiety and depression. However, there are few studies to date in physical health. In two large-scale trials in cardiac rehabilitation patients, adding MCT to rehabilitation was associated with improved anxiety, depression, and traumatic stress symptoms (28, 29). Pilot studies in cancer patients suggest that MCT is feasible and acceptable as a treatment approach (30). Consistent with the use of MCT to treat anxiety and depression in physical health, metacognitive beliefs have been found to be positively and significantly associated with psychological distress across a range of physical illnesses (31, 32).

The cognitive and metacognitive models lead to a different focus in therapy. A cognitive therapist challenges the reality of negative thoughts and beliefs about illness/injury as a means of treating anxiety, depression, and adjustment symptoms. Alternatively, an MCT therapist focuses on regulating worry, rumination, and attention and helps the patient revise their metacognitive beliefs irrespective of the content of thoughts.

Whilst previous research in BPRS patients has explored emotions following BPRS injuries (33) and how these relate to the development of specific psychiatric outcomes, such as PTSD (34), less is known about the cognitive and metacognitive experiences of BPRS patients. An exploration of such experiences can support an evaluation of the extent to which patients’ thoughts and feelings can be described by the CBT and MCT models. With this objective in the current study, we conducted open-ended semi-structured qualitative interviews to explore and understand the distress and thoughts of BPRS patients and to subsequently conceptualise their transcripts from the perspectives of the cognitive and metacognitive models to explore implications for treatment.

## Methods

### Ethical approval

This research was conducted as part of the first authors’ Clinical Psychology Doctorate. Ethical approval was gained from Greater Manchester Central Research Ethics Committee North West (REC reference: 21/NW/0050; IRAS ID: 289258).

### Patient and public involvement

Members of the University of Manchester’s Community Liaison Group (CLG) were consulted during the planning stage of this study. They provided valuable feedback on patient-facing documentation and the interview topic guide.

### Sample

Participants were recruited from the BPRS Psychology service at Wythenshawe Hospital in Manchester, UK. Inclusion criteria for the study was that patients must be (1) under the management of the BPRS Psychology team, (2) aged 18 years or older, (3) at least 1 month post BPRS injury, and (4) competent in English language skills (able to read, understand, and complete questionnaires in English). Patients were excluded if they met one or more of the following criteria: (1) had a cognitive impairment that precluded the ability to provide informed consent or the ability to participate, (2) were acutely suicidal, (3) were actively experiencing a psychotic episode, (4) had a current drug or alcohol dependence, (5) had ongoing deliberate self-harm, and (6) had dementia or learning difficulties. There were no restrictions placed on the size or type of the injury and no upper limit to how long ago the injury occurred.

### Materials

#### Demographic questionnaire

A demographic questionnaire includes age, sex, relationship status, highest level of education, current employment status, current living arrangements, current and past mental health background (i.e., current mental health rating on a visual analogue scale, past and present treatment), and details of their burn or reconstructive surgery.

#### Symptom outcome measures

To gain a description of the sample and of psychological distress symptoms at the time interview, participants completed the following measures:

*Patient Health Questionnaire-9* [PHQ-9; (35)]. The PHQ-9 is a nine-item measure designed to assess symptoms of depression in primary care settings. Each item corresponds to the DSM-IV criteria and is scored between 0 (not at all) to 3 (nearly every day). Total scores range from 0 to 27 representing mild (5–9), moderate (10−14), moderately severe (15−19), or severe depression (20−27). The scale demonstrates good reliability and validity (36).

*Generalised Anxiety Disorder-7* [GAD-7; (37)]. The GAD-7 is a brief seven-item scale to assess symptoms of generalised anxiety disorder in primary care. Each item is scored between 0 (not at all) and 3 (nearly every day) with total scores between 0 and 21 representing mild (5–9), moderate (10–14), and severe anxiety (15–21). The scale demonstrates good reliability and validity (38).

*Impact of Events Scale—Revised* [IES-r; (39)]. The IES-R is a 22-item measure designed to assess symptoms of PTSD. Patients are asked how distressing they have found each item in the previous 7 days and are scored between 0 (not at all) and 4 (extremely). Total scores range from 0 to 88 with scores of 24 or more indicating PTSD is a concern and scores of 33 or above indicating probable PTSD. The IES-r has been shown to have good reliability and validity in burn victims and motor vehicle accident survivors (40, 41).

### Procedure

Clinicians at the BPRS Psychology team routinely asked service users about research involvement. Interested patients completed a Consent to Contact form to allow the research team to contact potential participants to discuss the study further, screen for eligibility, and arrange a time to complete informed consent. Verbal consent was provided and recorded over university-approved video-conferencing software. Participants were given the option of completing the questionnaires over videocall, telephone, or via email.

The first author (JTB) conducted nine interviews, with a further two being conducted by another trainee clinical psychologist. Interviews were conducted in a conversational style over video conferencing software due to the COVID-19 pandemic limiting face-to-face research activity. All participants were reminded that questions were optional, that there were no right or wrong answers, and that they could pause or stop the interview at any time without giving a reason. An interview guide using open questions and prompts facilitated the interviews. Each participant was asked about their injury and mental health since the injury. They were also asked about their thoughts and adjusting to life after their injury. Their opinions on any psychological care they received and what ideal care might look like for someone in their position was also explored and will be reported elsewhere. Prompts were used to elicit further information relevant to the cognitive and metacognitive models.

Interviews were audio/video recorded and a transcript generated using the built-in function of the video conferencing software. Transcripts were reviewed and corrected against the recording by JTB and anonymised. NVivo (42) was used to manage data analysis.

### Data analysis plan

Interviews were analysed using thematic analysis [TA; (43)]. Analysis was conducted following the six phases of thematic analysis (43) to explore the emotional distress and mental reactions experienced by BPRS patients. JTB familiarised themselves with the data by listening to recordings and reading the transcripts several times. Themes were identified through inductive coding and discussed with LC.

To assess the extent to which a cognitive or metacognitive model provided a parsimonious account of the experiences of BPRS patients, a more deductive approach was used. JTB, LC, and AW identified key aspects of both the cognitive and metacognitive models *a priori* to use as a framework. For the cognitive model, 10 cognitive distortions were prespecified as codes, whereas for the metacognitive model, misdirected attention/control (e.g., hypervigilance, worry) and meta-beliefs (both positive and negative) were prespecified codes.

## Results

### Participant overview

In total, 14 patients provided informed consent to take part in the research; however, three patients withdrew following consent resulting in 11 patients (79%) taking part in the interview. Reasons for not taking part in the interview after providing informed consent were too busy to take part ( n = 1 ), unable to find a time that suited both researcher and participant ( n = 1 ), and no reason provided ( n = 1 ). **Table 1** provides an overview of participant characteristics. Participants were between 24 and 70 years ( mean=44.45, SD=10.10 ), predominantly women, n = 9 (82%), White British, n = 10 (91%), and worked full time, n = 6 (55%). Seven people (64%) reported a burn injury: five were thermal burns (four thermal, one contact), one was a chemical burn, and one a laser burn from a cosmetic procedure. The remaining four (36%) participants reported plastic reconstructive surgery as their primary intervention, with three having a deep inferior epigastric perforator flap (DIEP) surgery with full breast reconstruction and one having reconstruction following a boating accident. Of the 11 patients who took part in the interview, 7 (64%) reported that the event occurred more than 1 year ago, and 4 (36%) reported the event occurred 6 months to 1 year ago. Interviews lasted on average 63.68 min ( SD=18.19 ).

**Table 1:** Summary of demographic and physical health information for interviewed participants.

ID	Age (gender)	Relationship	Education^a^	Employment	Living	Ethnicity	Injury type	Time since index event	Comments
01	70 (f)	Married	University degree	Retired	Homeowner	White British	Burn	>1 year	Thermal burn to face
03	48 (f)	Married	University degree	Full time	Homeowner	White British	Reconstructive	>1 year	DIEP surgery
04	62 (m)	Separated/ divorced	GCSEs, GCEs, or O-levels	Full time	Homeowner	White British	Burn	>1 year	Thermal burns to face and arms
05	29 (m)	Single	Postgraduate	Student and part time	Homeowner	North African/British	Burn	6 month to 1 year	Acid burn—face, head/neck
06	34 (f)	Single	University degree	Full time	Homeowner	White British	Burn	>1 year	Laser burn to face
07	53 (f)	Married	University degree	Part time (14.5)	Homeowner	White British	Reconstructive	6 months to 1 year	DIEP surgery
08	48 (f)	Married	A-levels/BTECH	Full time	Homeowner	White British	Burn	6 months to 1 year	Thermal burn to lower body
10	47 (f)	Married	University degree	Full time	Privately renting	White British	Reconstructive	>1 year	DIEP surgery
12	24 (f)	Single	A-levels/BTECH	Unemployed	Living with family	White British	Reconstructive	6 months to 1 year	Boating injury
13	24 (f)	Single	A-levels/BTECH	Full time	Living with family	White British	Burn	>1 year	Contact burn to legs
14	50 (f)	Single	A-levels/BTECH	Unemployed	Living with family	White British	Burn	>1 year	Thermal burn

a Highest level of education attained at time of providing consent.

GCSE, General Certificate of Secondary Education; GCE, General Certificate of Education; BTEC, Business and Technology Education Council; DIEP, Deep Inferior Epigastric Perforator Flap.

Patients’ mental health is summarised in **Table 2**. The sample most frequently rated their mental health as 7 out of 10 ( range: 4−9 ), with lower scores indicating poorer mental health. Scores for depression (PHQ-9 mean=8.45, SD=4.76 ), anxiety (GAD-7 mean=7.91, SD=4.59 ), and PTSD (IES-R mean=29.64, SD=21.99 ) were all around the clinical cut-off scores for the respective measures. A majority of patients ( n=8, 73% ) were currently receiving mental health support.

**Table 2:** Summary of mental health information for interviewed participants.

ID	Diagnosis	Self-perceived mental Health status^a^	Current support	Past support	PHQ-9	GAD-7	IES-R			
							INT	AVD	HYP	Total
01	None	8	None	None	2	1	5	4	1	10
03	None	4	Therapy	Therapy	5	7*	6	6	2	14
04	None	9	None	None	0	3	0	1	0	1
05	None	5	Therapy	None	12*	7*	13	20	15	48*
06	Depression Anxiety	NA	Therapy Medication	Therapy Inpatient	10*	12*	12	9	8	29
07	None	7	Therapy	Therapy Medication	10*	6	12	4	5	21
08	None	7	Therapy Medication	Therapy Medication	11*	12*	24	14	21	59*
10	None	7	Therapy	Therapy	15*	9*	NA	NA	NA	NA
12	None	7	Therapy	None	5	3	20	21	17	58*
13	PTSD	7	None	Therapy	10*	11*	14	18	5	37*
14	Depression Anxiety PTSD	5	Therapy	Therapy	13*	16*	23	10	16	49*

a Visual analogue scale of current mental health at time of providing consent, whereby lower scores indicate poorer mental health.

NA, missing information; PHQ, Patient Health Questionnaire-9; GAD, Generalised Anxiety Disorder-7; IES-r, Impact of Events Scale—revised; INT, intrusion subscale of IES-r; AVD, avoidance subscale of IES-r; HYP, hypervigilance subscale of IES-r.

*Above clinical cut-off: PHQ-9 ≥ 9; GAD-7 ≥ 7; IES-r ≥ 33.

### Understanding patients’ emotional distress and reactions following a burn or reconstructive surgery

Three themes were identified when aiming to understand the distress that BPRS patients experience. These were as follows: (1) the broad range of feelings, (2) the level of engagement in repetitive negative thinking and the diversity of concerns that patients describe, and (3) the various coping strategies patients used as ways to control their thoughts and feelings.

#### Theme 1: Broad range of feelings

Patients experienced a range of feelings including “anger, guilt, sadness, and insecurity” (P06). A pattern did not emerge between the type of BPRS injury and feelings experienced, and all patients described experiencing a range of feelings.

Low mood ( n=9 ) and anxiety ( n=8 ) were the most common feelings described. Patients described feeling “incredibly low and very tearful” (P03), that they felt they were different, felt isolated or alone, and that “nobody really understood” (P10) what they were going through. They described feeling anxious, which was described as being fuelled by a sense of “vulnerability and fear” (P01) since their injuries.

Patients commented about the anger they experienced following their injury ( n=7 ). Patients were angry “that [it] happened” (P12) in the first place, at their lack of control over the situation, and at the extent and consequences of their injuries (P12: “I’m angry [ … ] I can’t use my arm and can’t go back to work”). One patient also spoke about how anger kept them focussed on the accident: “I was really angry all the time because most of the time I used to just think about the accident, it was all I thought about” (P12).

More than half of the patients described feeling guilty in the aftermath of their injury ( n=6 ). Patients reported feeling guilty about the “ripple effect through a family” (P14) who “all suffer too” (P07). Other patients, such as P06, said they felt “guilty for getting upset” about their injury because “in comparison to what happens to some people, it’s absolutely nothing.”

Although less common, patients also described feeling as though they had changed since their injury and feeling grief and loss ( n=4 ) about who they used to be as a person (P08: “you do, kind of, mourn the person that you used to be”).

#### Theme 2: Engagement in repetitive negative thinking and the diversity of concerns

All patients described engaging in repetitive negative thinking, namely, rumination and worry. Both worry and rumination focused on a range of concerns. Rumination commonly focused on why the event happened, their role in the event, and what it meant about them as people. Alternatively, worry predominantly focused on what others thought of them and their injuries, about the healing process of their injuries, and whether they would regain function, or get back to what they could do prior to their injury.

##### Rumination

All patients reported engaging in rumination ( n=11 ). This was primarily focussed on why the event had happened to them, that they “must of done something wrong” (P12) or questioning what they had done to “deserve this” (P03). Other concerns were around what could have happened “if the burns had gone any further” (P04) and what the potential consequences of that would be. Patients that had opted for surgical interventions to prevent or remove cancer reported an inner conflict between justifying why they made the decision to have the operation and questioning it (P10: “I still kind of think ‘what have I done to myself?’, which is bizarre when I know why I’ve done it”). Patients described how they were “constantly thinking [and] dwelling” which led to feelings being “bottled in” (P03). Although rumination was intended to reduce arousal, patients commented that it often had the opposite effect making them “feel worse” (P12) and that it led to a “spiralling that happens in my mind” (P05).

##### Worry

All patients reported engaging in worry ( n=11 ). Patients’ worrying covered a wide range of concerns. The majority of BPRS patients ( n=7 ) shared worries about their appearance; however, these manifested in different ways. Some shared worries about what they looked like after their injury (P01: “how can I live looking like this?”) and how others’ perceived them, saying “you can see them looking at it” (P05) and concerns that “no one’s ever gonna find me attractive” (P06). However, P03 and P07 who had both had the DIEP surgery to prevent or remove cancer both spoke about how it was the fact people “can’t see that there’s anything wrong” (P03) that led to worrying about being “knock[ed] into” (P03) as there was a fear this could cause pain and delay healing.

Almost all patients ( n=10 ) described worrying about their injury (i.e., whether it would heal, level of functioning, engagement in future activities). P14 worried their “physical changes” would stop them “escaping a fire in the pub or the club. P12 shared that for months after an injury to their arm, they worried that it was “never gonna work again,” and P01 recalled worrying about whether they would “be able to get back to work” and whether they were “ever going to run again.” Patients also worried about the likelihood of a similar accident or injury happening again in the future and “catastrophising about what might happen now to me” (P08).

Patients who had the DIEP surgery reported misinterpreting bodily symptoms, which led to worry that their cancer was returning. P07 described worrying whether a twinge in their back was “normal back pain” or “a tumour in my spine.” P03 worried about the effects that activities would have on them; if their stomach hurt, they would worry they had “done something to damage” themselves. These concerns were not shared by burn patients.

#### Theme 3: The various coping strategies used to control thoughts and feelings

Patients used a range of coping strategies to modify negative thoughts and attempt to control their thoughts and feelings. Distraction techniques (i.e., keeping busy, focussing on other tasks, watching television) were used by most patients ( n=9 ) to keep their “minds occupied” as a means of “not thinking about my accident” (P04). Others engaged in exercise in an attempt to keep themselves physically busy to avoid distressing thoughts. Some ( n=8 ) went further and engaged in active thought suppression, either trying to “ignore” thoughts (P08) or push them away. Other patients ( n=6 ) dealt with distressing thoughts by trying to replace them by “think[ing] of something positive” (P12).

Patients also spoke about avoidance ( n=9 ) and the range of reasons that motivated this. P01 avoided loud noises and the smell of smoke because it “brought it all back” (i.e., thoughts and feelings of their accident). P05 avoided going to new, busy, or unfamiliar places because of fear of being attacked again, and P03 spoke about “stepping aside massively to try and stay away from people” because of a fear of the pain and potential damage it could cause to their healing injuries. Patients also noted that they covered up more in front of their partners or family members and avoiding mirrors because of not liking how they looked; P14 spoke of their heart “beating through my chest” when they “caught a glimpse” of themselves in the mirror.

Fewer patients spoke about being hypervigilant ( n=4 ); however, it played an important role in feeling safe for those that did engage in it. P05 described being “very careful of where I step [ … ], who I speak to, [and] what I speak about” for fear of being attacked and that it helped them to prepare to “escape” if needed. P14, too, spoke of “constantly being on my guard for the next thing”.

### Can the underpinning concepts of the cognitive and metacognitive models be elicited from BPRS patients’ accounts of their psychological experiences since their injury?

The second aim was to explore whether concepts underpinning the cognitive and metacognitive models can be found in BPRS patients’ accounts of their psychological experiences since their injury to explore the fit of the models.

#### The cognitive model and BPRS patients’ accounts

Ten cognitive distortions were identified *a priori* by the co-authors. **Table 3** provides an overview of the cognitive distortions selected, the number of patients who evidenced each distortion, and examples of such distorted thinking that exemplify how these manifested in patient talk. Cognitive distortions were identified in every patient, with most patients exhibiting multiple distortions.

**Table 3:** Key constructs of the cognitive model with examples from interviews.

Construct	Description	Example from interviews
NATs	Spontaneous thoughts that occur in everyday cognition	“If somebody knocks into me then I know that’s really going to hurt” (P03) “It’s not fair” (P07) “I’m a lost cause” (P13) “I look grotesque” (P14)
Catastrophising	Negative predictions about the future, with no consideration of other possible outcomes	“Would I look anywhere near normal ever again?” (P01)
Overgeneralisation	Reaching a negative conclusion that goes beyond the present situation	“You start assuming that everyone’s out there to get you” (P05)
Dichotomous thinking	A situation is viewed in only two categories	“I’ve not had real cancer” (P03)
Mind reading	Belief that others are thinking negatively about oneself, without considering more positive possibilities	“They’re not really interested” (P07)
Labelling	A negative label is assigned to oneself and/or others, without consideration of other evidence	“I’m a bit of a basket case psycho those days” (P07)
Imperatives	A fixed idea of how oneself or others should behave, and when these expectations are not met, a negative outcome is overestimated	“I must be a terrible person for this to have happened to me” (P03)
Discounting the positives	Positive experiences or qualities do not count	“It is extremely good outcome in terms of burns, but it’s still to me a Scarface” (P01)
Magnification/minimisation	When a person or situation is evaluated, the negative is magnified and/or the positive is minimised	“In comparison to what happens to some people, it’s absolutely nothing” (P06)
Prediction/fortune telling	Predicting a negative outcome without realistically considering the actual odds of that outcome	“Life isn’t going to be worth living looking like this” (P01)
Emotional reasoning	Something is regarded as true because it is felt strongly, evidence to the contrary being ignored	“I feel like an idiot” (P07)
Intermediate	Rules, attitudes, and assumptions that people have about themselves, others, and the world around them	“I need to be proactive and look after my health” (P07)
Core	Peoples’ beliefs about themselves, others, and the world around them that are deeply entrenched and regarded as absolute truths	“I’m a walking example of a burns patient” (P01)

NAT, negative automatic thought.

Catastrophising often related to the permanence of the current situation, patient’s abilities, or appearance and was closely linked to the injury or event. For example, P01 questioned whether they would “look anywhere near normal again,” and P06 voiced similar concerns, stating “what if I don’t ever look how I want to look?”. P08 recalled sitting near a fire and thinking “it’s going to explode.” In a similar way to catastrophic thinking, overgeneralisation was often related directly to the injury or event. P06 “won’t be going for a beauty treatment again” as this was the setting their burn occurred in and they were “not keen” on a laser treatment recommended by BPRS surgeons citing that lasers were “why I’ve got [the injury] in the first place.” Patients who were taking part in the research after surgery to remove or prevent cancer reported overgeneralising the frequency people talked about cancer with “somebody always on the telly [ … ] or somebody in your group or who’s got [cancer]” (P07).

BPRS patients often reported thinking that they knew what other people thought of them (mind reading). This was almost always related to concerns about patients’ appearance. Burn patients often spoke about how distressing it was that their injury was visible: P01 said “what they will think of my face” and that “people can see there is a problem,” P12 recalled thinking “people were looking at my scars,” and P13 assumed people thought about them as “the girl with the burn scars.” On the other hand, those who had had surgery that was not visible feared that “people can’t see that there’s anything wrong” (P03) and would not know to steer clear; otherwise, contact could exacerbate their injuries and delay healing.

Imperatives are a fixed idea of how oneself or others should behave, and examples of this distortion often related to self-blame as follows: “I must be a terrible person for this to have happened to me” (P01), “if I hadn’t of done that, then it wouldn’t have happened” (P12), and “it’s my fault” (P13). Those who had the DIEP surgery particularly seemed to think in this way due to the elective element of the surgery; for example, P07 said “I feel like I’ve done this to myself,” and P03 said it was “my fault that this has happened.”

Some of the concerns shared seemed unrealistic and were clear examples of cognitive distortions. For example, “you start assuming that everyone [is] out there to get you” from P05 is a clear example of overgeneralisation. However, there were numerous examples where concerns may have reflected the patients’ clinical reality. Several patients voiced concerns about other people noticing their injury and reacting to it. For example, a concern from P01 that other people could see their burn injury and the mask they wore was true as it was “in your face, literally” (P01). P10 stated that they “just looked different,” while perhaps not as obvious as they thought, this could be considered objectively true after their DIEP surgery removing breast tissue. Further, P07 had concerns that their “risk of ongoing cancers is pretty high” may be justified; they had been diagnosed with breast cancer on three occasions despite not meeting the criteria for any clinical risk factors, and the risk of recurrence is higher in those who have already been diagnosed, approximately 30% for breast cancer (44). There were also concerns about the implications of physical changes; for example, P14 shared a concern that they “wouldn’t be able to get out fast” if there was a fire in a pub or club, which may be justified due to the extent and impact of their injuries on mobility.

Some statements made by patients could be interpreted in a number of ways and could be categorised as more than one cognitive distortion. For example, when talking about their injured arm, P12 stated “it’s never gonna work again,” which was coded as an overgeneralisation, but could easily have been interpreted as catastrophic thinking, a prediction, or not seen as a distortion at all if this turned out to be clinically true. Similarly, P01 discounted the positive aspect of their scar healing as being objectively “good” but then also labelled herself as a “Scarface.” It was at times hard to determine which cognitive distortion was the best “fit” to the statement being made.

**Table 4** demonstrates that BPRS patients’ accounts exhibit thoughts consistent with symptoms of a range of problem-specific models. For example, P01 exhibited distressing cognitions pertinent to both PTSD and Social Anxiety (i.e., re-experiencing and avoidance, and strong opinions about what other people think of them; see **Table 4**); however, they did not score above the clinical threshold on any measure at screening and reported never receiving any psychiatric diagnoses. Similarly, P08 exhibited thoughts that would indicate the use of numerous problem-specific models including PTSD, depression, and GAD (see **Table 4**). They were hypervigilant for threat, avoidant of reminders of their accident, distressed by worries about a range of concerns, and reported having little hope about coping with events when they felt low in mood.

**Table 4:** How problem-specific CBT models might fit patients accounts.

Problem-specific model	Description	Evidence from interviews
PTSD	A sense of current, serious threat arising from: 1) negative appraisals of the trauma, and 2) memory disturbances with strong associative memories. Often resulting in episodes of re-experiencing the trauma (e.g., flashbacks), hypervigilance, and avoidance (45)	*Re-experiencing* “The bangs and the noises and you think “it’s all happening again” (P01) “The more stronger the flashback, the more real the sensations feel to me” (P05) “I dream about someone or something that was associated with it” (P12) “I panicked, and I freaked out, I saw it and I just went into meltdown” (P13) “I’d doze off in the day and wake up screaming” (P14)
		*Hypervigilance* “I was very … very wary of where I go … I need to be extremely careful of who I’m speaking with, who’s walking towards me, what’s behind me, what’s the setting, can I escape somehow?” (P05) “I’m very much more nervous and anxious around things” (P08) “Because of what’s happened and constantly being on my guard for … for the next thing” (P14)
		*Avoidance* “I won’t go near the little camping stove because that’s what it that blew up” (P01) “I couldn’t even use the oven because the heat would make me physically shake” (P08)
Depression	Negative Automatic Thoughts driven and maintained by negative beliefs about the self (12, 13). Termed the Cognitive Triad thoughts tend to group into: 1) thoughts of being inadequate, 2) trying leads to failing, and 3) no hope for the future (14)	“I think when I do let them happen [negative thoughts] it possibly makes me feel worse” (P06) “I’ve been so down [ … ] you do lose hope, you definitely do lose it” (P14) “I don’t think you’ll ever, ever fully get to point where you go “oh everything’s brilliant” you know?” (P06) “I’m really not able to cope with it” (P08)
Social anxiety	Misinterpretation of own thoughts and concerns about appearance and social functioning that forms a strong impression of how they appear to others and seeing social situations as a threat (46)	“What will they think of my face” “do they not know who I am or is it so bad that they are just too embarrassed to talk to me?” (P01) “I’m always doing this [touching silicone tape over scar] when I’m talking to people because I’m almost doing it to make them know I’m aware that that’s there”; “no one’s ever gonna find me attractive [ … ] I’m never going to find me attractive” (P06) “People were looking at my scars” “people would see me differently because of what happened” (P12) “I do feel I’m going to get judged a lot” (P13)
GAD	A heightened preoccupation with threat coupled with an underestimation in their ability to cope with it results in a cycle of worry and an intolerance of uncertainty (47, 48)	“What didn’t help was not knowing what I was going to look like [ … ] would I look anywhere near normal ever again?” (P01) “I’m constantly worrying or thinking about this” (P05) “Catastrophizing about what might happen now to me”; “you’re consistently wanting your brain to just stop doing that but it’s on a roll, it kinda just … it runs away with itself” (P08) “Worrying too much or something will lead to the worst catastrophe” (P13)

NA, not applicable as not assessed.

Concepts underpinning the cognitive model could be elicited from BPRS patients’ accounts of their psychological experiences since their injury. There were multiple examples of each type of pre-specified cognitive distortions and multiple cognitive distortions in each patient’s account. Additionally, patient talk could be categorised into disorder-specific models (e.g., social anxiety, health anxiety, PTSD, depression). Some concerns appeared to be based on the patient’s clinical reality making it hard to determine whether these were examples of biased thinking, and some patient talk appeared to fit more than one problem-specific model.

#### The metacognitive model and BPRS patients’ accounts

All aspects of the CAS (i.e., repetitive negative thinking, inflexible attention, threat monitoring) could be elicited from BPRS patients’ accounts of their psychological experiences post-injury (see **Table 5**). It was also possible to elicit a range of maladaptive metacognitive beliefs, which BPRS patients stated interfered with regulation of their thoughts and feelings.

**Table 5:** Key constructs of the metacognitive model with examples from interviews.

Construct	Subtype	Description	Examples from interviews
Perseverative negative thinking	Worry	Repetitive negative thinking about potential threats/danger	“No one’s ever gonna find me attractive” (P06) “[Is my arm] never going to work again?” (P12) “[Am I] ever going to run again” (P01) “[Will I] be able to get back to work?” (P01)
	Rumination	Repetitive negative thinking about past events	“What have I done to myself?” (P10) “[I’m] constantly thinking [and] dwelling” (P03)
Threat monitoring	N/A	A heightened state of vigilance where the individual constantly scans for signs of potential danger or problems	“Constantly being on my guard for the next thing” (P14) “[I have to be] very careful of where I step [ … ], who I speak to, [and] what I speak about” (P05)
Inflexible attention	N/A	Feeling unable to disengage attention from negative thoughts, symptoms, events	“It’s constantly there physically, so it’s been constantly there psychologically as well” (P10) “Once I start worrying about one thing, then I’ll start worrying about something else, and something else” (P06)
Dysfunctional coping behaviours	N/A	Behaviours intended to reduce distress or reduce overthinking that are counterproductive	“Well I avoid fire. Anything to do with fire I really, I really don't like.” (P01) “Think[ing] of something positive” (P12)
Meta beliefs	Positive	Beliefs about the usefulness of worry, rumination, importance, and danger of thoughts	“[Worrying] has some kind of benefit” (P03) “Worrying and analysing can also help me focus” (P13) “A little bit of a worry might help me get my thoughts into place” (P07)
	Negative	Beliefs about the uncontrollability, importance, and danger of thoughts	“[Worrying is] “seriously dangerous [and] destroys your mind” (P01) “My thoughts are in control” (P13) “[My brain] runs away with itself” (P08)

As noted under research question one theme two (Repetitive Negative Thinking), patients all engaged in rumination and worry. Patients ruminated on why the event had happened, their role in it, and what it meant for them as a person. The content of worries was wide and varied; there was repetitive engagement in worries about appearance, what other people thought about them, their injury (i.e., whether it would heal, level of functioning, engagement in future activities), and about what kind of threat bodily sensations might represent. Such engagement in the CAS was described as an attempt to “deal with it” (P05); however, patients described it as having the opposite effect and generating “a lot of questions that I don’t have answers for” (P06). Others acknowledged, too, that the process was “stopping [them] from moving on” (P14) and led to their minds “spiralling” (P05).

Patients described experiences that may indicate a lack of flexibility in their attention. Patients described feeling unable to shift their attention away from negative thoughts or feelings. Patients described how the persistent focus on threat (or pain) meant that their distress was maintained; “it’s constantly there physically, so it’s been constantly there psychologically as well” (P10). This sometimes manifested in avoidance and hypervigilance as noted under research question one, theme three (Coping Strategies).

Patients endorsed two common maladaptive negative metacognitive beliefs: *worrying is uncontrollable* and that it is *harmful or dangerous*. Patients noted that they often felt out of control of their thinking, stating that “there wasn’t much I could do about it” (P05) and that their brain “runs away with itself” (P08). P06 said “once I start worrying about one thing, then I’ll start worrying about something else, and something else” and that it “just happens at random” highlighting that they “don’t feel particularly in control.” This sentiment was echoed by P13, saying “my thoughts are in control.” Patients also spoke about how worrying was “seriously dangerous” and that it “destroys your mind” (P01). Two patients noted that they believed that excessive worrying could “lead to suicide” (P01) and that their neighbour had “worried [themselves] to death” (P01). Both P06 and P14 shared that they believed worry to be “destructive” and P06 added it was “almost like a form of self-harm”. As in some of these examples above, some patients worried about worry itself (meta-worry), with P13 saying that “worrying too much” can make the thing more likely to happen and will “lead to the worst catastrophe.”

Patients also spoke about positive metacognitive beliefs such as worrying being a helpful process that allowed them to be more prepared. P03 described worry as being “protective” and helping avoid “end[ing] up back in hospital.” P05 said worry “has some kind of benefit” because it led to them “being extra-cautious and wary,” which could help them avoid another potential attack. P13 believed that “worrying and analysing can also help me focus,” and P07 expressed that “a little bit of a worry might help me get my thoughts into place.”

The key concepts of the metacognitive model can be elicited from BPRS patients’ accounts of their psychological experiences after their injury. There were multiple examples of all aspects of the CAS (i.e., repetitive negative thinking, inflexible attention, threat monitoring) as well as examples of both positive and negative metacognitive beliefs. Repetitive negative thinking encapsulates a diverse range of concerns shared by BPRS patients without having to reality check the content.

## Discussion

The first aim of this study was to explore and understand the psychological experiences of BPRS patients, that is, the feelings, thoughts, and coping behaviours since their injury. The second aim was to explore whether concepts underpinning the cognitive and metacognitive models could be elicited from BPRS patients’ accounts of their psychological experiences since their injury.

Patients described a broad range of feelings, such as sadness, anxiety, anger, and grief, and many patients described experiencing more than one in the aftermath of their injury. Repetitive negative thinking (i.e., worry and rumination) was common across all patients with the content varying considerably. There was a range of maladaptive coping strategies described as an attempt to manage thoughts and feelings. These included thought suppression, distraction, avoidance, and positive thinking in an attempt to control thoughts and emotions. These results are in line with those of McPhillips et al. (49) who evaluated emotional distress in cardiac rehabilitation patients and found that patients experienced a range of worries, believed that worrying was uncontrollable and harmful, and utilised maladaptive coping strategies (i.e., reassurance seeking). Further, the use of strategies, such as thought suppression and avoidance, has been shown before in burn patients (33) and have been posited at factors that may maintain distress and PTSD symptomatology in BPRS patients (34).

Concepts underpinning the cognitive and metacognitive models could be elicited from BPRS patients’ accounts of their psychological experiences since injury. The cognitive model makes a judgement as to whether the content of concerns is biased or distorted. There were multiple examples of all 10 pre-specified cognitive distortions in BRPS patients’ accounts. However, there were examples of thoughts that could not be classified as they may have been either distorted thinking or based on clinical reality. BPRS patients’ talk could be characterised as being consistent with symptoms of a range of problem-specific models.

The metacognitive model seeks to understand how much people engage in the CAS and hold negative and positive metacognitive beliefs about worry/rumination. There were examples of all aspects of the CAS such as repetitive negative thinking (i.e., worry and rumination), inflexible attention, and unhelpful coping strategies. Positive and negative metacognitive beliefs were also readily identifiable in BPRS patients’ accounts and could be identified irrespective of the content of negative thoughts.

The results suggest that both the cognitive and metacognitive models could be applicable within the BPRS patient population to help make sense of and modify patient experiences. Applying a deductive approach to patient talk suggested differences in the ease with which each model might accommodate the full range of patient experiences.

The cognitive model relies on engaging in discussion of the content of thoughts and finding distortions in such content. Some of the concerns reported by BPRS patients had a basis in clinical reality; topics such as appearance concerns, fear of cancer recurrence, and feeling at risk of another attack/accident could be realistic. Previous research on fear of cancer recurrence suggests that such concerns are often reported and “one of the top greatest concerns and the most frequently endorsed unmet need” (50). It has been highlighted that a purely cognitive approach (e.g., using cognitive restructuring) may not adequately address concerns of this nature (23). Behavioural methods of CBT may be important in these cases (51), and adaptations to CT/CBT have been suggested (23, 52), such as gaining additional information from health specialists and incorporating this into judgements about the likelihood of future events and using acceptance-based approaches if this does not work. Research into adaptations of this kind suggests that they are acceptable (53) and that they can be successfully implemented in primary care settings (54), although they may be described as “resource intensive” (p. 5). Further research is needed to determine the efficacy and cost effectiveness of adapting CBT for people with physical health concerns. A barrier to such an approach is knowing which adaptations are required and how they fit within a cognitive model. The metacognitive model does not focus on the content of concerns (i.e., *what* people worry about) and instead focusses on the mechanisms underlying *why* people worry. In contrast with CBT, MCT does not rely on judging thoughts as realistic/unrealistic or containing specific distortions. This means that patients do not have to share the content of their worries with practitioners, which could be a potential benefit. Furthermore, The BPRS population has high levels of trauma (7, 11), and making disclosures and focusing on the content of memories may in itself be distressing.

A feature of Beck (14) cognitive theory is the hypothesis that affective states can be discriminated based on the content of cognition, referred to as the content-specificity hypothesis, where distinct emotional disorders have distinct content in distressing cognitions. We found that many BPRS patients account fit problem-specific models of distress, including concerns consistent with PTSD, social anxiety, depression, and generalised anxiety-specific models. This is encouraging and suggests these models may be of benefit in this population. Several patients, however, spoke about concerns that could be considered symptoms for multiple psychological presentations, and each has distinct treatment approach based on cognitive content. For example, P01 shared concerns about their appearance and being judged by others as well as concerns about reexperiencing the event; this could fit into several distinct cognitive models and treatments (e.g., social anxiety and PTSD). A potential consequence is that therapists must be able to successfully determine a hierarchy of needs and target the most important need first. It is also possible that patients could require several distinct episodes of care to address all of their concerns. It is worth noting that this study did not set out with the intention to determine which problem-specific model would be most useful for BPRS patients and the interviews did not constitute a diagnostic interview. It is also worth noting that just because a patient shared concerns consistent with a particular presentation (e.g., social anxiety), it does not mean that they reached clinical threshold for this requiring treatment. Further research could utilise diagnostic interviews to clarify whether BRPS patients meet criteria for multiple diagnoses.

As MCT focusses on processes defined as the CAS and their regulation, rather than content, it is often referred to as being a transdiagnostic treatment that can deal with a wide range of psychological presentations without the need to modify the protocol. This means there would not be a decision about which model to apply to whom, in what sequence, and therefore opens up the possibility of efficient, less distressing, and group-based interventions across different presentations.

The approach to sample recruitment in the present study did not specify strict inclusion criteria. While this approach allowed more people to participate and offered a representative reflection of the BPRS psychology service being recruited from, it may have under-represented the distress and concerns that are held by this population. Despite some patients not scoring above clinical cut-off scores on any psychometric measure, they talked about challenging feelings, thoughts, and behaviours.

The current study has a small sample size, which could limit the generalisability of findings. Qualitative studies tend not to require sample sizes as large as those required in quantitative studies, and data saturation was reached with the sample that was recruited. However, it should be noted that there was minimal diversity within the sample; most participants were White British women, and therefore, this may have restricted the breadth of beliefs and experiences captured.

Qualitative studies are shaped by the researchers’ perspectives, and it should be noted that members of the research team are experts in both the cognitive and metacognitive models, but the team included the originator of the metacognitive model and MCT (AW). The first author was aware of this throughout the process, and the team worked to acknowledge and account for this potential bias by grounding the research in the narratives of the patients’ experiences and specifying aspects of both models *a priori* before examining interview transcripts.

## Conclusions

The psychological experiences of BPRS patients are varied with a wide range of feelings and negative thoughts, engagement in repetitive negative thinking and evidence of metacognitions in patient accounts. Key concepts underpinning both the cognitive and metacognitive models could be identified in BPRS patients’ accounts of their psychological experiences. The constructs of CBT were often ambiguous because of the highly variable and multiple contents of patients’ negative thoughts, some of which seemed realistic. The constructs of MCT might provide a simpler fit in accounting for patient experiences as all talk could be described by the concepts of the CAS and metacognitive beliefs, with little or no ambiguity.

CBT and MCT present alternative potential approaches to help patients manage psychological distress and adjustment following BPRS. Future studies should investigate the effectiveness of CBT or MCT as interventions.

## Data Availability

The data will be available upon request to the authors. This is in line with our ethical approvals.
